# Effects of vitamin D on insulin resistance and fasting blood glucose in pregnant women with insufficient or deficient vitamin D: a randomized, placebo-controlled trial

**DOI:** 10.1186/s12902-022-01159-4

**Published:** 2022-10-20

**Authors:** Zahra Mirzaei-Azandaryani, Sakineh Mohammad-Alizadeh-Charandabi, Elnaz Shaseb, Shamsi Abbasalizadeh, Mojgan Mirghafourvand

**Affiliations:** 1 Students’ research committee, Nursing and Midwifery Faculty, Social Determinants of Health Research Center, Tabriz University of Medical Sciences, Tabriz, Iran; 2 Department of Midwifery, Faculty of Nursing and Midwifery, Tabriz University of Medical Sciences, Tabriz, Iran; 3 Department of Clinical Pharmacy, Faculty of Pharmacy, Tabriz University of Medical Sciences, Tabriz, Iran; 4 Women’s Reproductive Health Research Center, Tabriz University of Medical Sciences, Tabriz, Iran; 5 Social Determinants of Health Research Center, Faculty of Nursing and Midwifery, Tabriz University of Medical Sciences, Tabriz, Iran

**Keywords:** Vitamin D, Fasting blood glucose, HOMA-IR, Pregnancy, Gestational diabetes mellitus, Depression

## Abstract

**Background:**

Gestational diabetes is one of the most common metabolic disorders during pregnancy. Some studies have reported the effect of vitamin D deficiency on the incidence of this disorder. Therefore, the purpose of the present study was to determine the effect of vitamin D supplementation on fasting blood glucose (FBG) levels, fasting blood insulin (FBI) levels and insulin resistance index (HOMA-IR) (primary outcomes) and symptoms of depression, musculoskeletal pain, frequency of gestational diabetes and the frequency of abortion (secondary outcomes).

**Methods:**

In this triple-blind randomized controlled trial, 88 pregnant women at 8–10 weeks of pregnancy who had the vitamin D of less than 30 ng/ml were randomly assigned to the vitamin D group (n = 44) and control group (n = 44) using block randomization. The vitamin D group received 4,000 units of vitamin D tablets daily and the control group received placebo tablets for 18 weeks. Independent t-test, Mann-Whitney U and ANCOVA tests were used to analyze the data.

**Results:**

After the intervention, there was no statistically significant difference between the two groups in terms of FBG (P = 0.850), FBI (P = 0.353), HOMA-IR (P = 0.632), mean score of depressive symptoms (P = 0.505), frequency of gestational diabetes (P = 0.187) and frequency of abortion (P = 1.000) and there was only a difference in terms of serum vitamin D level (P = 0.016) and musculoskeletal pain including knee pain (P = 0.025), ankle pain (P < 0.001) and leg pain (P < 0.001).

**Conclusion:**

Vitamin D could improve the musculoskeletal pain in pregnant women but couldn’t decrease FBG, FBI, HOMA-IR, depression symptoms score, incidence of GDM and abortion.

**Trial registration:**

Iranian Registry of Clinical Trials (IRCT): IRCT20120718010324N59. Date of registration: 4/11/2020. URL: https://en.irct.ir/user/trial/50973/view; Date of first registration: 21/11/2020.

## Introduction

Pregnancy changes affect glucose metabolism and insulin sensitivity [1]. If this disorder of blood glucose regulation occurs for the first time during pregnancy, it is called gestational diabetes. This disorder is one of the most common metabolic disorders during pregnancy [2, 3]. 1–14% of pregnant women in the world have gestational diabetes. According to the International Diabetes Federation, hyperglycemia occurs in one of every four pregnancies, and it is diagnosed as gestational diabetes in 90% of cases [4]. The annual incidence of gestational diabetes in the United States is 3–8%, in Australia 6–10%, in China 14% and in Iran 7% [5]. Gestational diabetes has many short-term and long-term complications for mother and baby, including preterm delivery, cesarean delivery, fetal overgrowth, neonatal hyperinsulinemia, hypoglycemia, hyperbilirubinemia, intrauterine fetal death, etc. [3, 6].

Vitamin D is a fat-soluble vitamin that comes in two forms, D2 and D3; Type D3 is made under the skin under the influence of the sun’s ultraviolet rays or is taken orally from seafood and egg yolks, and type D2 is obtained from yeast and edible mushrooms [7, 8]. Vitamin D less than 20 ng/ml is considered vitamin D deficiency. A dose of 20–30 ng/ml means vitamin insufficiency and amounts above 30 ng/ml are defined as normal [9, 10]. Over the past two decades, vitamin D deficiency has been identified as a pandemic. This deficiency is seen in 90% of the general population and in almost all age groups [11]. Pregnant women are more susceptible to this deficiency due to the increased need for micronutrients [12]. During pregnancy, the rate of vitamin D conversion to 25-hydroxyvitamin D does not change, but the rate of conversion of 25-hydroxyvitamin D to 1 and 25-dihydroxyvitamin D increases to large amounts until the 12th week of pregnancy, its amount doubles that of non-pregnant women [13]. In the United States, two out of every three pregnant women have vitamin D deficiency [12]. In Iran, 84% of pregnant women have been reported with vitamin D levels below 25 ng/ml [14].

The role of vitamin D in bone health has been well established, but it has been suggested that it may also play a role in the health of other tissues, including the regulation of blood glucose [15]. A number of studies have shown that vitamin D has a specific receptor on pancreatic beta cells [16–18]. Some studies have shown that insulin resistance is higher in pregnant women with vitamin D deficiency [18, 19].

Vitamin D is also active in the hypothalamus-pituitary-adrenal pathway [20]. In parts of the brain, such as the thalamus, hypothalamus, amygdala, and some motor neurons, there is a high density of vitamin D receptors and this issue suggests that vitamin D has an effect on the sensory pathways, some motor pathways and the autonomic system. Therefore, some symptoms of depression such as fatigue, mood regulation, motor function and pain may be related to vitamin D deficiency [21]. A review study also shows that there is a relationship between low levels of vitamin D and the onset of depressive symptoms during pregnancy and postpartum [22]. A clinical trial have shown a positive effect of vitamin D supplementation on improving depressive symptoms in depressed patients [23]. The homeostatic role of vitamin D and its role in the immune system and nervous system protection can explain the biological link between the depression and vitamin D [24]. Some studies showed there is an association between level of 25(OH)D and musculoskeletal pain [25–27]. Hormonal and physiologic changes that occur in pregnancy, increase the risk of musculoskeletal pain [28]. The studies conducted by Heath et al. and Knutsen et al. showed vitamin D supplementation lead to improve muscle function [25, 29].

Considering the effect of vitamin D deficiency on maternal and neonatal outcomes including the increased risk of gestational diabetes [30–34] and its positive association with depression [22] and musculoskeletal pain [25–27], vitamin D supplementation appears to be valuable for those with vitamin D deficiency [35]. There are many studies assessing the effect of vitamin D supplementation in pregnant women with gestational diabetes who had vitamin D deficiency [18, 19, 36], but other studies have been conducted among pregnant women without gestational diabetes [15, 37]. Also, all studies have been conducted on pregnant women with a gestational age of more than 12 weeks and results of the trials [15, 18, 19, 36, 37] are inconsistent. Therefore, this study aimed to determine the effect of vitamin D supplementation on fasting blood glucose (FBG) levels, fasting blood insulin (FBI) levels and insulin resistance index (HOMA-IR) (primary outcomes) and symptoms of depression, musculoskeletal pain, frequency of gestational diabetes and the frequency of abortion (secondary outcomes) in pregnant women with a gestational age of 8–10 weeks.

## Methods

### Study design and participants

This triple-blind randomized controlled clinical trial was performed on 88 pregnant women referring to health centers in Hamadan-Iran from January 2021 to November 2021. In this study, participants, data collector and analyst did not know the type of intervention received.

Inclusion criteria were pregnant women with a gestational age of 8–10 weeks with a level of vitamin D less than 30 ng/ml and a body mass index of less than 30 kg/m^2^. Exclusion criteria included people with a history of diabetes or currently having diabetes, people with a history of polycystic ovary syndrome or currently having the disease, people with thyroid and parathyroid disorders, people with a previous history of musculoskeletal pain, people with a history of delivering a macrosomic baby, history of vitamin D intake in the last three months and scores above 12 on the Edinburgh Postpartum Depression Scale (EPDS).

### Sampling

Sampling for this study was done in eight centers out of 60 health centers in the city of Hamedan after obtaining the code of ethics from the ethics committee of Tabriz University of Medical Sciences (ethics code: IR.TBZMED.REC.1399.759) and registering the study in the Iranian Registry of Clinical Trials (code: IRCT20120718010324N59). Sampling was conducted in densely populated and socio-economically different centers. Contact information of potentially eligible pregnant women was extracted from the integrated health system (SIB system) and they were contacted. During the telephone call, the objectives and method of the study were briefly explained, and they were evaluated according to some inclusion and exclusion criteria. If they were eligible and also willing to participate in the study, they were invited to refer to the health center at a specified time. In the face-to-face meeting, the objectives and method of the study were fully explained. In case they were willing to participate in the study, written informed consent was obtained. The EPDS was completed by individuals, and blood samples were taken from individuals with a depression score of 12 or less to assess vitamin D levels. Some of their blood samples were frozen in the laboratory to be used to measure other study variables as needed. In women with vitamin D level less than 30 ng/ml, a questionnaire on socio-demographic information and obstetric history and a musculoskeletal checklist were completed using an interview method and their frozen blood sample was investigated in terms of FBI, FBG and also HOMA-IR was evaluated.

### Randomization

Participants were randomly assigned to vitamin D and placebo groups using block randomization with block sizes of 4 and 6 and 1:1 allocation ratio. Assignment sequence was performed by a person not involved in the sampling and data analysis. To conceal the allocation, vitamin D and placebo were placed in similar opaque containers numbered from 1 to 88. The containers were given to the participants in order of entering the study.

### Intervention

The treatment group received 4,000 units of vitamin D daily and the other group received placebo. Vitamin D was prepared by Zahravi Pharmaceutical Company (Tabriz-Iran) and placebo by Dana Pharmaceutical Company (Tabriz-Iran). It should be noted that acute vitamin D poisoning usually occurs in the consumption of doses above 10,000 units per day and chronic poisoning occurs after consumption of doses above 4,000 units per day for a long time [38].

The intervention started at 8–10 weeks of pregnancy and continued until the end of 26 weeks of pregnancy. All pregnant women were followed up monthly and evaluated for side effects. It should be noted that all participants took a multivitamin supplement that contains 400 units of vitamin D daily from the end of the 16th week of pregnancy. From this time on, the protocol for taking the pills (whether placebo or vitamin D) was changed to taking it on Saturday to Thursday (not taken on Friday).

### Instruments

In this study, sociodemographic and obstetric questionnaire, EPDS, laboratory tests checklist, musculoskeletal pain checklist, side effects checklist and drug checklist were used to collect data. At the end of the 26th week of pregnancy, the EPDS and the musculoskeletal pain checklist were completed again by all participants and blood samples were taken for FBG, FBI, HOMA-IR, Vitamin D, and OGTT.

#### Sociodemographic and obstetric characteristics questionnaire

The sociodemographic and obstetric characteristics questionnaire included questions about age, level of education, number of pregnancy and childbirth, job, income, life satisfaction, etc. The validity of this questionnaire was determined through content and face validity.

#### Edinburgh postnatal depression scale (EPDS)

The EPDS is used to measure pregnancy and postpartum depression and was developed by Cox et al. in 1987 [39]. This tool consists of ten four-choice questions. In some questions, the options range from low to high (items 1, 2, and 4) and in some, from high to low (items 10, 9, 8, 7, 6, 5, 3). The options for each question have a score from zero to three based on the intensity of the symptom, and the final score a person earns is obtained from the points of the answers to a total of ten questions, which can vary from zero to 30. Mothers who score above the 12 threshold have varying degrees of depression. The validity and reliability of this scale was confirmed by Montazeri et al. (2007) in Iran [40].

#### Laboratory tests checklist

Serum vitamin D level, FBG, FBI, HOMA-IR index and Oral Glucose Tolerance Test (OGTT) for each individual were recorded in a special checklist. FBG concentration was considered after 8–14 h of fasting, provided that they have a normal diet and normal physical activity for at least the last three days [37]. In 2013, the World Health Organization and the International Diabetes Association recommended a two-hour OGTT single-level test with 75 g of oral glucose to screen and diagnose gestational diabetes at 28 − 24 weeks of gestation for all pregnant women. Accordingly, in case fasting plasma glucose was 92 mg/dl or higher, or one-hour glucose was 180 mg/dl or higher, or two-hour glucose was 153 mg/dl or higher, gestational diabetes was diagnosed [41]. Vitamin D less than 20 ng/ml was considered as vitamin D deficiency. A dose of 20–30 ng/ml means vitamin insufficiency and amounts above 30 ng/ml are defined as normal [9, 10]. One of the important indicators in measuring insulin resistance is the HOMA-IR index, which is measured using a fasting blood sample. This index was calculated by multiplying the fasting glucose concentration (nmol/l) by the fasting insulin concentration (ml/l) divided by 22.5 [18].

#### Musculoskeletal pain checklist

In this checklist, three items including knee pain, ankle pain and leg pain were examined by the answers yes and no. This checklist was developed by research team and its validity was determined through content and face validity.

#### Drug checklist

In this checklist, daily intake of vitamin D3 was recorded by each participant.

Side effects checklist.

In this checklist, any side effects that occurred in the study and its severity were recorded.

### Sample size

The sample size in this study was calculated based on both fasting glucose and insulin resistance index using G-Power software. Based on the results of the study by Soheilykhah et al. (2013) [18] on the variable of insulin resistance index and taking into account M_1_ = 1.54 (mean insulin resistance index before intervention), M_2_ = 1.15 (assuming 25% reduction due to intervention), SD_1_ = SD_2_ = 0.69, One-sided α = 0.05 and Power = 80%, the sample size was calculated equal to 40 people in each group. However, considering the 10% drop, the final sample is 44 people in each group. Based on fasting glucose variable and considering M_1_ = 78.0 (average plasma level of fasting glucose before intervention), M_2_ = 70.2 (assuming 10% reduction due to intervention), SD_1_ = SD2 = 8.21, One-sided α = 0.05 and Power = 95%, it was calculated equal to 25 people in each group. Considering 10% drop, the final sample is 28 people in each group. Due to the fact that the sample size calculated based on the variable of insulin resistance index was higher, so the final sample size was considered to be 44 people in each group.

### Statistical analysis

Data were analyzed using SPSS software version 24. Kolmogorov-Smirnov test was used to check the normality of the data. Fasting blood glucose levels before and after the intervention, depression score before the intervention and one-hour and two-hour glucose tolerance tests had normal distribution. Vitamin D and fasting blood insulin levels before and after the intervention, insulin resistance index before and after the intervention and depression score after the intervention had abnormal distribution. Mean (SD) were reported for normally distributed data and median (IQR) for abnormally distributed data. Chi-square, chi-square for trend, independent t-test and Fisher’s exact test were used to evaluate the homogeneity of the study groups in terms of sociodemographic and obstetric characteristics. To compare the data with normal distribution between the two groups, we used independent t-test and ANCOVA with adjustment of baseline values. For abnormally distributed data, change score (post-intervention value-baseline value) was calculated and the normality of the change score was assessed using Kolmogorov-smirnov test. Only the change of fasting blood insulin had normal distribution. Then, independent t-test was used to compare the two groups in terms of the data with normal distribution, ​​and Mann-Whitney U test for data with abnormal distribution,

## Results

### Baseline characteristics

**Fig. 1:**
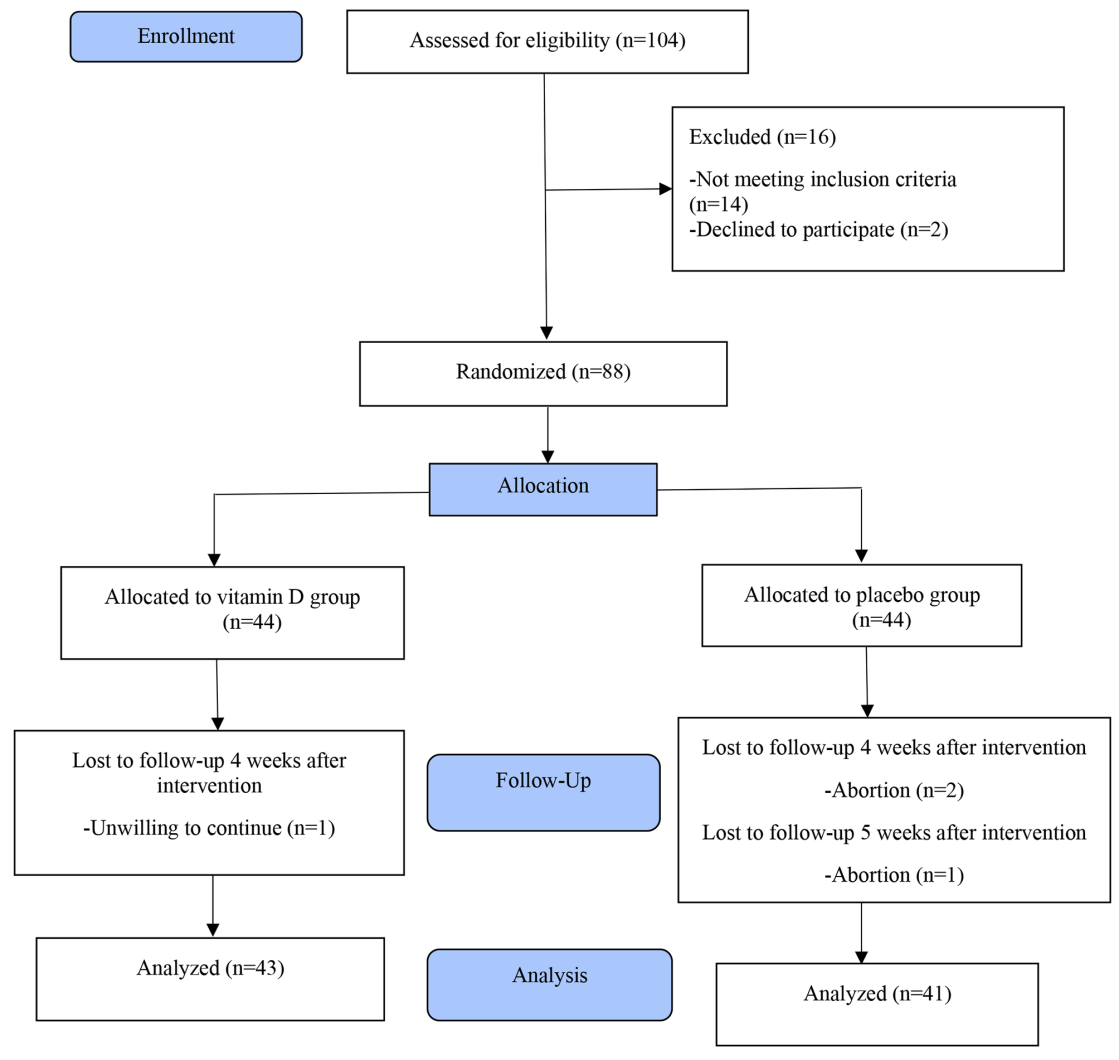
Study flow chart

Out of 104 pregnant women with gestational age of 8–10 weeks, 14 were excluded from the study due to lack of meeting the inclusion criteria (six due to body mass index above 30 kg/m^2^, two due to history of macrosomic baby, three due to history of polycystic ovaries, three due to a history of gestational diabetes in a previous pregnancy) and two cases were excluded due to unwillingness to participate in the study. 84 cases participated and stayed until the end of the study and were analyzed (43 in the vitamin D group and 41 in placebo group). In the placebo group, three people were excluded from the study due to spontaneous abortion and in the vitamin D group, one person was excluded from the study due to lack of willingness for cooperation (Fig. 1).

There was no statistically significant difference between the two groups in terms of sociodemographic and obstetric characteristics (P > 0.05), except for spouse’s occupation (P = 0.019) that this difference was adjusted using ANCOVA test. Mean (SD: standard deviation) of the age of participants in the vitamin D group was 25.6 (6.08) and it was 27.5 (6.25) years in the placebo group. 11.4% of the participants in the vitamin D group and 20.5% of the participants in the placebo group had university education (Table 1).

**Table 1:** Sociodemographic and obstetrics characteristics in study groups

Variable	Vitamin D group (n = 44)	Placebo Group (n = 44)	P-value
	**Mean (Standard deviation)**	**Mean (Standard deviation)**	
**Age (year)**	25.63 (6.08)	27.54 (6.25)	0.151*
**BMI (kg/m²)**	24.52 (2.97)	24.21 (3.43)	0.653*
	**Number (Percent)**	**Number (Percent)**	
**Education level**			0.119^†^
Elementary	5 (11.4)	5 (11.4)	
Secondary	17 (38.6)	11 (25)	
High school	5 (11.4)	8 (18.2)	
Diploma	12 (27.3)	11 (25)	
University	5 (11.4)	9 (20.5)	
**Job**			1.000^‡^
Housekeeper	43 (97.7)	44 (100)	
Working at the home	1 (2.3)	0	
**Husband education level**			0.733^†^
Elementary	5 (11.4)	4 (9.1)	
Secondary	17 (38.6)	16 (36.4)	
High school	5 (11.4)	7 (15.9)	
Diploma	12 (27.3)	11 (25)	
University	5 (11.4)	6 (13.6)	
**Husband job**			0.019^‡^
Self employed	29 (65.9)	31 (70.5)	
Employed	1 (2.3)	7 (15.9)	
worker	14 (31.8)	6 (13.6)	
**Income sufficiency**			0.832^†^
More than sufficient	0	0	
Sufficient	22 (50)	23 (52.3)	
Less than sufficient	22 (50)	21 (47.7)	
**Gravid**			0.903^‡^
One	19 (43.2)	18 (40.9)	
Two	15 (34.1)	17 (38.6)	
Three and more	10 (22.7)	9 (20.5)	
**Parity**			0.826^‡^
No	21 (47.7)	21 (47.7)	
One	14 (31.8)	16 (36.4)	
Two and more	9 (20.5)	7 (15.9)	
**Abortion history**			1.000^§^
No	39 (88.6)	40 (90.9)	
One	5 (11.4)	4 (9.1 )	

*Independent t-test; †Chi-square for trend; ^§^ Fishers Exact Test; ^‡^ Chi-Square

### Primary outcomes

Before the intervention, there was no statistically significant difference in FBG (Mean Difference (MD): -0.27; P = 0.888), FBI (P = 0.160) and HOMA-IR index (P = 0.148) between the two groups. After the intervention, there was no significant difference in terms of FBG (MD: 0.37; P = 0.850) according to ANCOVA test. Also no significant difference was observed between the two groups in terms of FBI (P = 0.353) and HOMA-IR index (P = 0.632) (Table 2).

**Table 2:** Comparison of Vitamin D, FBG, FBI, HOMA-IR, Depression score and OGTT between study groups

Variable	Vitamin D			Placebo			P-value
	**Mean (SD** ^**‡**^ **)**		**Median (per 25 to 75** ^**§**^ **)**	**Mean (SD** ^**‡**^ **)**		**Median (per 25 to 75** ^**§**^ **)**	
**Vitamin D level**							
Before intervention	14.86 (5.90)		14.50 (9.52 to 18.47)	17.50 (6.65)		17.20 (12.07 to 22.92)	0.065•
After intervention	32.17 (13.20)		30.40 (25.00 to 35.00)	26.51 (9.99)		25.70 (17.75 to 32.30)	0.002•
**FBG**							
Before intervention	87.09 (7.63)		87.00 (83.00 to 92.75)	87.36 (10.26)		87.00 (83.00 to 92.75)	0.888*
After intervention	81.97 (8.90)		81.00 (77.00 to 85.00)	82.37 (8.46)		82.00 (75.50 to 87.00)	0.850^†^
**FBI**							
Before intervention	6.38 (3.63)		5.69 (3.80 to 8.50)	8.63 (7.58)		7.70 (4.17 to 10.35)	0.160•
After intervention	7.24 (5.11)		6.19 (3.83 to 9.14)	8.55 (5.97)		7.04 (4.68 to 10.10)	0.672*
**HOMA-IR**							
Before intervention	24.43 (13.75)		21.41 (14.69 to 33.60)	33.87 (29.97)		26.61 (16.23 to 42.88)	0.148•
After intervention	27.29 (26.63)		21.01 (12.94 to 33.34)	29.85 (23.90)		22.62 (14.13 to 35.90)	0.637•
**OGTT (60 min)**							
After intervention	133.28 (22.74)		130.50 (115.00 to 150.00)	127.31 (31.57)		117.00 (100.00 to 152.00)	0.354*
**OGTT (120 min)**							
After intervention	110.18 (18.58)		111.00 (95.00 to 123.00)	107.80 (24.47)		106.00 (89.00 to 126.00)	0.639*
**Depression score**							
Before intervention	9.11 (5.16)		8.00 (5.00 to 13.00)	8.88 (4.29)		8.00 (5.25 to 12.00)	0.822*
After intervention	8.62 (6.47)		7.00 (4.00 to 12.00)	8.87 (5.07)		8.50 (5.00 to 12.00)	0.404•

•Mann-Whitney U; * Independent Samples Test; ^†^ANCOVA; ^‡^ Standard Deviation; ^§^ Percentile 25 to Percentile 75

In variables with abnormal distribution, all analysis after intervention was conducted on change score (post-intervention value-baseline value)

### Secondary outcomes

There was no statistically significant difference before the intervention in terms of mean score of depressive symptoms (MD: 0.23; P = 0.822) and serum vitamin D level (P = 0.065). After the intervention, there was no significant difference between the two groups in terms of mean rank score of depressive symptoms (P = 0.505) and a statistically significant difference in terms of vitamin D level (P = 0.016) (Table 2).

Based on chi-square test, before the intervention, there was no statistically significant difference in musculoskeletal pain including knee pain (P = 0.938), ankle pain (P = 0.730) and leg pain (P = 0.314) between the two study groups. There was a statistically significant difference between the two groups after the intervention in terms of all three variables of knee pain (P = 0.025), ankle pain (P < 0.001) and leg pain (P < 0.001). Based on independent t-test, there was no statistically significant difference between the two groups in terms of OGTT 60 min (MD: 5.97; P = 0.354) and OGTT 120 min (MD: 2.38; P = 0.639). Also, according to Fisher’s exact test, there was no statistically significant difference between the two groups in terms of frequency of gestational diabetes (P = 0.187) and frequency of abortion (P = 1.000) (Table 3).

**Table 3:** Comparison of musculoskeletal pain, gestational diabetes mellitus and abortion between study groups

Variable	Vitamin D group	Placebo Group	P-value
	**Yes (Number/Percent)**	**Yes** **(Number/Percent)**	
**Knee pain**			
Before intervention	15 (34.9)	15 (34.1)	0.938^‡^
After intervention	6 (14.0)	14 (35.0)	0.025^‡^
**Ankle pain**			
Before intervention	7 (16.3)	6 (13.6)	0.730^‡^
After intervention	2 (4.7)	15 (37.5)	< 0.001^‡^
**Leg pain**			
Before intervention	15 (34.9)	11 (25.0)	0.314^‡^
After intervention	3 (7.0)	29 (72.5)	< 0.001^‡^
**Gestational diabetes incidence**	1 (2.56)	4 (11.42)	0.180^§^
**Abortion incidence**	0	3 (6.81)	1.000^§^

^‡^ Chi-Square; ^§^ Fishers Exact Test

In terms of side events, one person in the vitamin D group and two people in the placebo group reported nausea. One person in the placebo group reported headache, one in the vitamin D group, and three in the placebo group reported constipation.

## Discussion

Findings of the present study showed that taking vitamin D supplement of 4000 units daily from 8 to 10 weeks of pregnancy in pregnant women with vitamin D level less than 30 ng/ml, although increased vitamin D level and improved musculoskeletal pain, it did not have a significant effect on FBG, FBI, insulin resistance index, depressive symptoms, gestational diabetes and miscarriage.

In many of previous trials, the effect of vitamin D has been evaluated on pregnant women with gestational diabetes [18, 19, 36, 42]. In studies by Mojibian et al. (2015) and Yap et al. (2014) the effect of vitamin D has been investigated on pregnant women without gestational diabetes but in the both trials, vitamin D supplementation was started after 12 weeks of pregnancy [15, 37]. Due to the importance role of vitamin D in embryonic cytotrophoblastic cells and organogenesis, and for the proper formation of the placenta, in this trial, intervention was started in the first trimester of pregnancy.

The results from study performed by Yap et al. (2014) on pregnant women with mean 14 weeks of pregnancy and vitamin D levels less than 32 ng/ml are consonant with the results of the present study. In this study, taking 5,000 units of vitamin D daily until 28 − 26 weeks of gestation had no effect on improving blood glucose levels and insulin resistance; however, it increased vitamin D levels [15].

A systematic review and meta-analysis of randomized controlled trials showed that vitamin D supplementation in pregnant women with gestational diabetes improves glycemic control [43]. Another systematic review and meta-analysis of randomized controlled trials has been conducted in pregnant women with or without gestational diabetes with no subgroup analysis by having or not having gestational diabetes. The results showed that vitamin D supplementation decreased HOMA-IR index in mothers [44]. The inconsistency between the results of the above studies and present study may be due to differences in participants and study design. In the present study, women without gestational diabetes have been evaluated.

Vitamin D probably has a beneficial effect on insulin function by stimulating insulin receptor expression, thereby increasing the insulin response to glucose transport into the cell [45]. The indirect effect of vitamin D on insulin secretion is a calcium-dependent process [46], and vitamin D plays this role by affecting the penetration of calcium. Inadequate calcium intake or inadequate vitamin D level may change the balance between extracellular and intracellular calcium levels in pancreatic beta cells, which may interfere with normal insulin secretion [47]. Simultaneous consumption of calcium and vitamin D is also useful in improving glucose metabolism. Therefore, the probable reason for conflicting results in studies could be the different nutritional status of the participants [47, 48]. It should be said that some inflammatory biomarkers such as TNF-α, interleukin 6 increase in diabetes. On the other hand, vitamin D affect immune system and decreasing inflammatory biomarkers. The results of observational studies also provide conflicting evidence as to whether low serum levels of 25-hydroxy vitamin D (25 (OH) D) are associated with gestational diabetes [49–52].

In the present study, vitamin D intake had no effect on improving the mean score of depressive symptoms. However, a study by Vaziri et al. (2016) on non-depressed (healthy) pregnant women showed that daily intake of vitamin D 2000 from 26 to 28 weeks until childbirth reduced depressive symptoms during pregnancy [53]. The results of the review of observational studies by Aghajafari et al. (2018) also showed a relationship between depression and serum levels of vitamin D [22]. The studies by Okereke et al. (2020), Omidian et al. (2019), Mason et al. (2016) performed on non-pregnant population [23, 54, 55]. Meanwhile, mood disorders happen commonly during pregnancy [55]. So the results of these studies can’t be generalized to pregnant women. In Vaziri et al. (2016)’study, depression score has been evaluated in different weeks of pregnancy compare with our study. Also the difference in the results of the above studies and present study may be due to differences in participants, duration of treatment, gestational age, received dose amount and type of tool used, too.

The findings of the present study showed that daily intake of 4000 units of vitamin D in pregnant women had no effect on reducing the incidence of abortion. However, some studies have shown a relationship between vitamin D and the incidence of miscarriage [56–58]. A trial study conducted by Samimi et al. (2016) showed that in people with spontaneous miscarriage, consumption of 400 units of vitamin D daily reduced the rate of miscarriage [59]. Given that most of the studies conducted in this field were observational, and the frequency of abortions in this study was examined as a secondary outcome, therefore, stronger clinical trials with larger sample sizes are recommended.

The present study showed that vitamin D supplementation improved musculoskeletal pain in pregnant women. A randomized controlled trial by Lozano-Plata et al. showed that vitamin D supplementation in patients with fibromyalgia improves their pain [60]. No clinical trial in this topic has been done on pregnant women. Although a systematic review of randomized clinical trials by Lombardo et al. (2022) showed that vitamin D supplementation in patients with chronic musculoskeletal pain may reduce pain in these people. In this review participants were in all genders and ages with different health status that subgroup analysis has not been done [61].

In this study, no significant side effects were observed in the study groups. No side effects have been reported in some clinical trials [15, 54, 62–66]. The acute toxicity with vitamin D happens when people intake above 10,000 units of doses per day that increases serum concentrations to above 150 ng/ml and also chronic toxicity occurs after consuming amounts of more than 4000 units per day for a long time [38].

### Strengths & Limitations

Adherence to all principles of clinical trial, including random allocation, concealment of allocation, and blinding of participants and outcome assessor were among the strengths of this study that minimized bias resulting from selection, performance and detection. The use of standard and valid questionnaires was another strength of this study. This study was performed on women with insufficient or deficient vitamin levels, therefore the results cannot be generalized to pregnant women with normal vitamin D level. The small number of samples was another limitation of this trial. Therefore, it is recommended that future studies be performed with a larger sample size. In our study, the nutritional status of participants was not assessed. It is also suggested to control the nutritional status of the participants in future studies, so that different nutritional status of the participants does not distort the results. Serum levels of vitamin D should also be measured in pre-pregnancy care, and intervention should be started before pregnancy in people with insufficient or deficient levels of vitamin D.

## Conclusion

Based on the findings of this study, daily intake of 4000 units of vitamin D in the first trimester of pregnancy in women with vitamin D levels less than 30 ng/ml for 18 weeks has no effect on improving fasting glucose and fasting insulin levels, insulin resistance, depression score, the incidence of gestational diabetes and miscarriage. However, it was effective on increasing vitamin D level and improving musculoskeletal pain. Also, this dose of vitamin D in pregnant women with vitamin D level below 30 ng/ml was safe and no side effects were observed.

## Data Availability

The datasets generated and/or analysed during the current study are not publicly available due to limitations of ethical approval involving the patient data and anonymity but are available from the corresponding author on reasonable request.

## Abbreviations

FBG: Fasting Blood Glucose.
FBI: Fasting Blood Insulin.
HOMA-IR: Insulin Resistance Index.
IRCT: Iranian Registry of Clinical Trials.
EPDS: Edinburgh Postpartum Depression Scale.
OGTT: Oral Glucose Tolerance Test.
SD: Standard Deviation.
95% CI: 95% Confidence Interval.
